# Supplementary material for: Cascading hazards of a major Bengal basin earthquake and abrupt avulsion of the Ganges River
Source: Nat Commun. 2024 Jun 17;15:4975. doi: 10.1038/s41467-024-47786-4 (PMC11183210; doi:10.1038/s41467-024-47786-4)
Supplement: Supplementary file 1 — Supplementary Information [file 41467_2024_47786_MOESM1_ESM.pdf]

Supplementary Information for "Cascading hazards of a major Bengal basin earthquake and abrupt avulsion of the Ganges River"

Elizabeth L. Chamberlain[1,2,3*], Steven L. Goodbred[2*], Michael S. Steckler[3], Jakob Wallinga[1], Tony Reimann[4], Syed Humayun Akhter[5,6], Rachel Bain[2], Golam Muktadir[7], Abdullah Al Nahian[5], FM Arifur Rahman[5], Mahfuzur Rahman[5,8], Leonardo Seeber[3], Christoph von Hagke[9]

1. Soil Geography & Landscape group and Netherlands Centre for Luminescence dating, Wageningen University, Wageningen, The Netherlands
2. Department of Earth & Environmental Sciences, Vanderbilt University, Nashville, TN, USA
3. Marine & Polar Geophysics, Lamont-Doherty Earth Observatory, Columbia University, Palisades, NY, USA.
4. Mathematics & Natural Sciences, University of Cologne, Cologne, Germany.
5. Department of Geology, University of Dhaka, Dhaka, Bangladesh.
6. Bangladesh Open University, Gazipur, Bangladesh
7. Department of Environmental Science, Bangladesh University of Professionals, Dhaka, Bangladesh
8. Department of Oceanography, Noakhali Science and Technology University, Bangladesh
9. Department of Environment & Biodiversity, Geology and Physical Geography, University of Salzburg, Salzburg, Austria

*corresponding authors: liz.chamberlain@wur.nl and steven.goodbred@vanderbilt.edu

Supplementary Methods

1. Characterization of seismite features

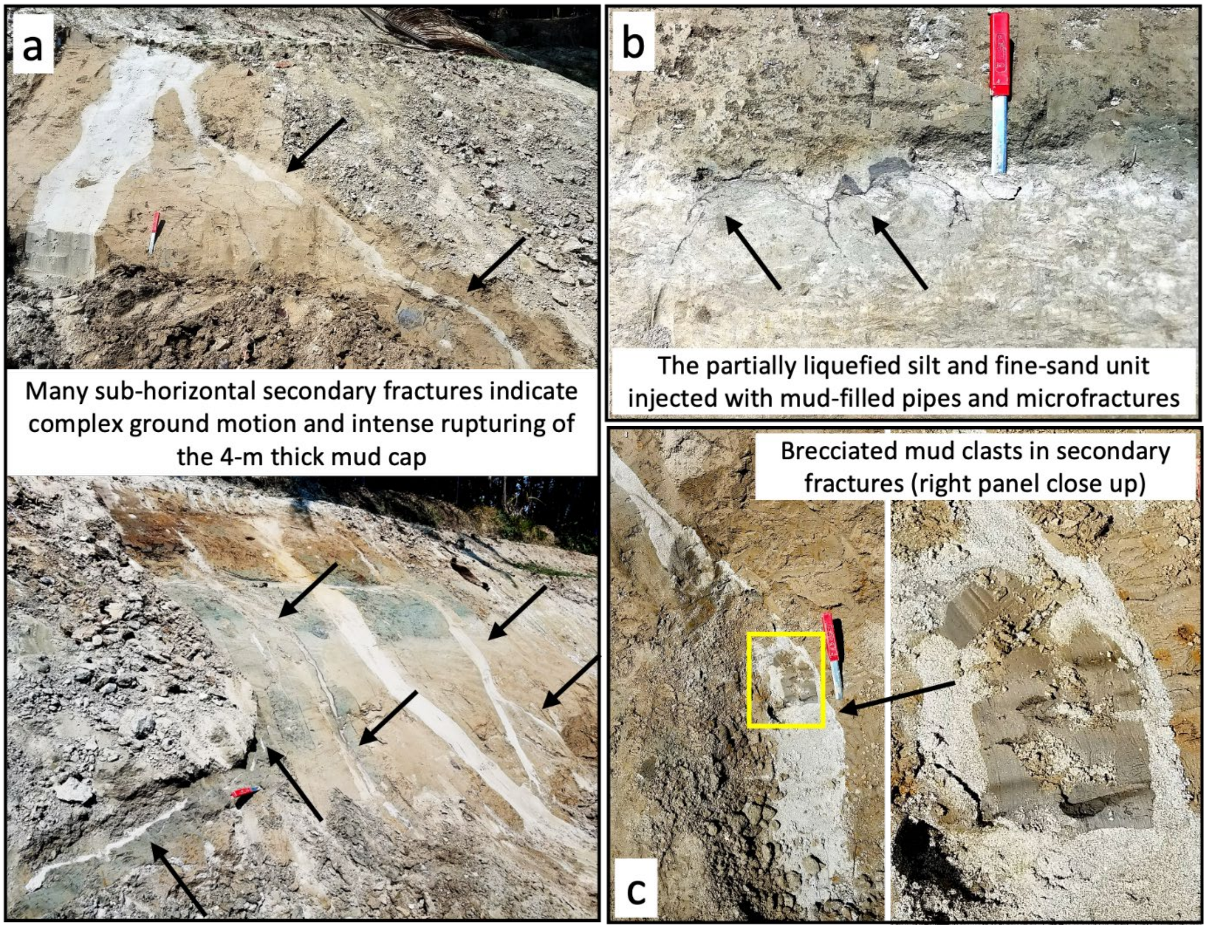

Fig. S1. Numerous and varied features related to sediment liquefaction and the sand injectites that are exposed within the excavated pond. Features shown here, in addition to those in Figure 5 of the main text, include: **a**, multiple subordinate fractures that bifurcate from main dike, **b**, liquefied silt and fine sand with deformed cross bedding and localized mud injections, and **c**, brecciated mud clasts in the subordinate fractures.

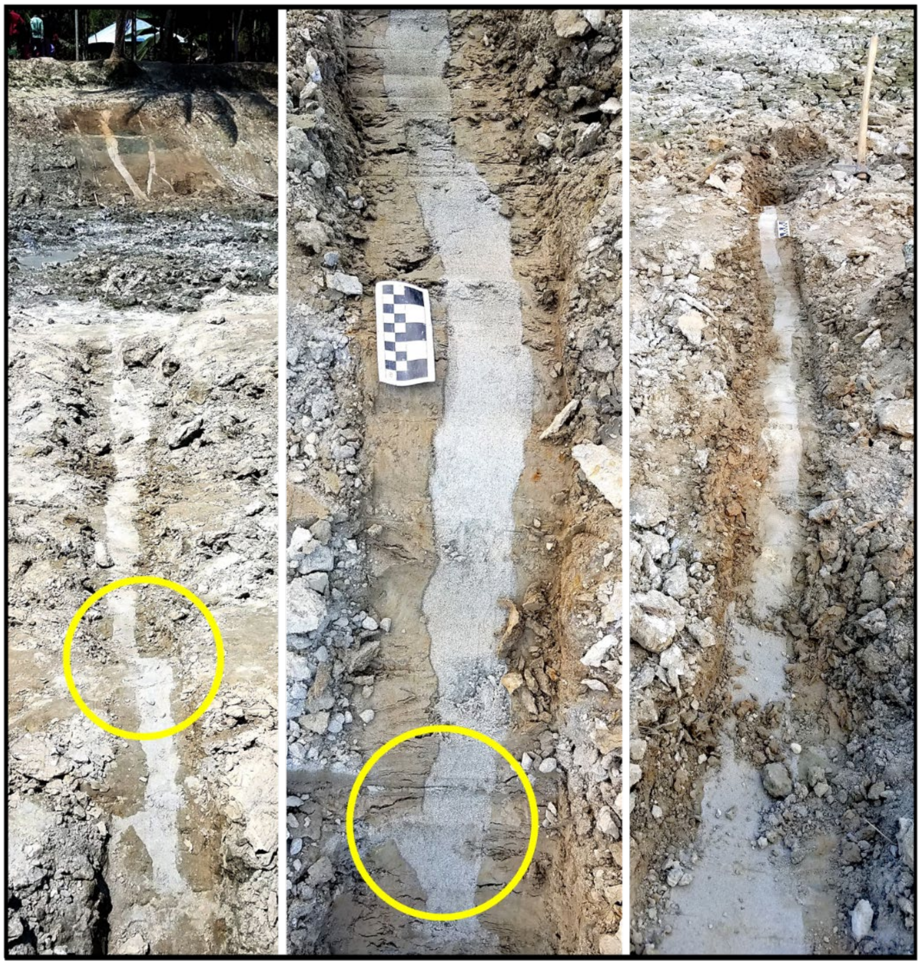

Fig. S2. Oblique views of the roughly linear sand-intruded fractures that extend across the excavated pond floor. Note local transverse offsets (yellow circles) that suggest fracturing by Love waves.

2. Downstream extent of the paleochannel belt

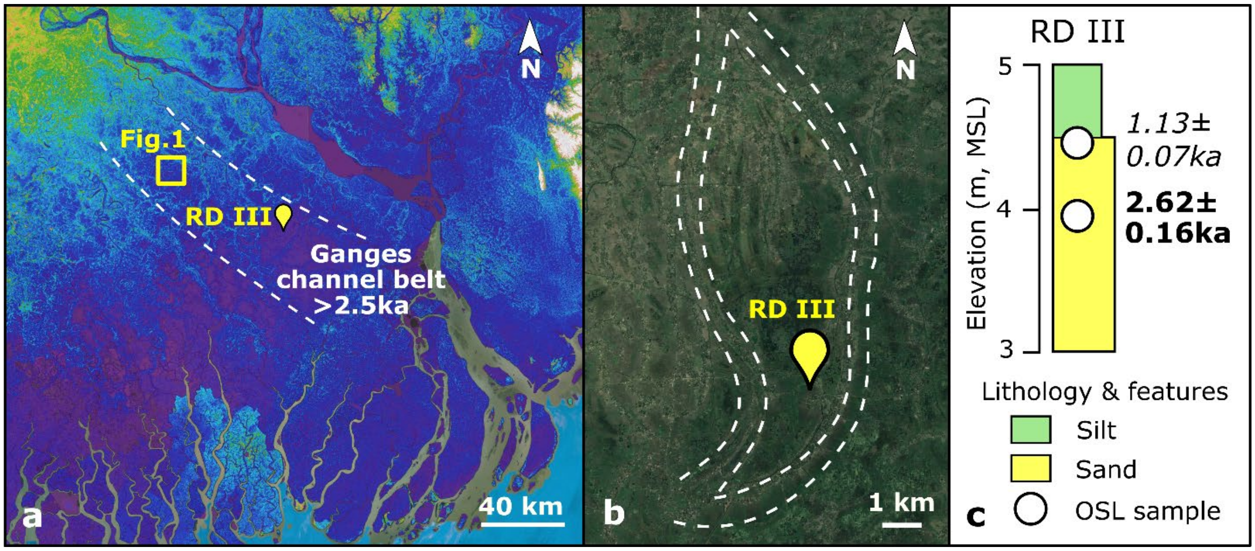

Fig. S3. a, Coring and dating at the Ramdia site (RD III), ~ 85 km southeast of the seismite and paleochannel locations (see Fig. 1), revealed the downstream location of the abandoned paleochannel belt. **b**, Surface morphology suggests a mid-channel bar top. **c**, Sandy deposits ~1 m below surface date to 2.62 ± 0.16 ka, which is contemporaneous with the 2.61 ± 0.11 ka age for the top of point-bar sands in the upstream channel (Fig. 2). Sands at the Ramdia site (RD III) were veneered by ~60 cm of bioturbated agricultural soil that yielded an unreliable luminescence age (shown in italics). Bulk strontium content confirmed that the sands were sourced by the Ganges River (see S3). All borehole and OSL dating details are reported in Supplementary Data 1-3.

3. Geochemical and grain-size measurements

Bulk strontium is a reliable indicator for Holocene sand provenance in the Bengal delta, with pure Brahmaputra deposits higher in strontium (>140 ppm) than those of the Ganges (<110 ppm), with intermediate values (110-140 ppm) generally characterizing mixed-source sediment[1]. To determine the provenance of the OSL samples, we measured bulk major and trace element concentrations on 20 g of dried bulk sediment using a portable XRF (Thermo-Scientific Niton XL3 Analyzer). We also measured the grain-size distribution of each sample by laser diffraction using a Malvern Mastersizer 3000 particle-size analyzer. Bulk strontium content and median grain size (D_{50}) values are reported in Supplementary Data 2.

The bulk strontium measurements ranged from 83 ± 3 to 98 ± 3 ppm, indicating that all paleochannel sand samples at the Magura (MA IV-1, MA IV-2, and MA V-1) and Ramdia sites (RD III-1 and RD III-2) were sourced by the Ganges River. These results, in conjunction with the bar-top sand ages, confirm that the sites define a former course of the Ganges River. All mud samples of from the Magura paleochannel sites (MA V-2) and adjacent seismite pond (MA I-2, MA I-3, MA I-4, and MA II-1) also yielded bulk strontium characteristic of Ganges river deposits, with values ranging from 64 ± 3 to 88 ± 3 ppm, although it should be noted that bulk strontium has not been robustly vetted for provenance of muds in this system. No mud samples were taken from the Ramdia site.

Bulk strontium results of sands at the seismite pond were more complex. The seismite sand dike sample (MA III-1) and shallowest sand sample (4.00-4.15 m below land surface) extracted by coring below the pond floor yielded bulk strontium values of 94 ± 3 and 93 ± 3 ppm respectively, well within the range of Ganges-sourced sediment. However, the two deeper sand samples (4.50-4.65 and 4.60-4.75 m below land surface), also collected by coring, yielded strontium values of 126 ± 3 and 116 ± 3 ppm respectively, indicative of mixed-source sediment of both the Ganges and Brahmaputra rivers. Mixing of sources can happen in several ways including mainstem river confluence, tidal reworking, or bioturbation. In this case, however, we suspect a different cause, with deeper Brahmaputra-sourced sediment being mixed upward into the overlying Ganges sediment during the liquefaction and sand injection. The mixing of coarser Brahmaputra sediments is also consistent with the median grain size of these two samples being greater than that of other shallow sands in the dataset that have a pure Ganges Sr signature (Supplementary Data 2).

4. Optically Stimulated Luminescence (OSL) dating

S4.1 Sample preparation. Samples were prepared for luminescence measurement under safe amber light conditions at Vanderbilt University, Nashville, USA, and etched and measured at the Netherlands Centre for Luminescence dating (NCL), Wageningen, NL. We focused on fine silt for this study, as this has proven to be the most effective grain-size fraction for dating Holocene-aged G-B deposits[2]. Our silt preparation techniques follow those of Chamberlain, et al. [2]. Silt was isolated from sieved washwater using settling velocities calculated with Stokes Law to obtain the 4-11 μm fraction of mud-rich samples and the 4-20 μm fraction of sand-rich samples (Supplementary Data 3). Chemical treatment was applied with 30% H_2O_2 to remove organics and 10% HCl to remove carbonates, yielding polymineral silt. A polymineral fraction containing quartz, feldspar, and heavy mineral grains was retained for polymineral multiple-signal single-aliquot regenerative dose (MS-SAR) measurement[3]. Up to 1 g of polymineral silt per sample (as available) was etched in 40 ml 31% fluorosilicic acid (H_2FSi_6) for ~ 168 hrs (one week) with twice-daily stirring to isolate the quartz fraction. Fluorosilicic acid was decanted and samples were rinsed three times with deionized water, then cleaned with 10% HCl for 1 hr and rinsed four times with deionized water. The efficacy the etch was verified with an IR depletion test[4]. The obtained purified quartz fraction was used for SAR measurement[5].

Silt was prepared for measurement by pipetting 0.1 ml of ~ 20 mg/ml polymineral or quartz silt suspended in acetone directly onto 10 mm-diameter stainless steel disk (roughly 10^6 grains). A description of the samples is provided in Supplementary Data 2.

S4.2 Dose rate determination. Bulk sediment was dried for 1-2 days in an oven at 50 °C to obtain water content (expressed as percent dry weight), burned overnight at 500 °C to obtain organic content, ground to homogenize the material, then mixed in wax and cast as pucks each containing ~157.5 g sediment. Pucks were stored for at least three weeks prior to measurement of radionuclide activity concentrations on a high-resolution broad-range gamma spectrometer at Wageningen University. Dose rates experienced by the silt grains were calculated as the sum of natural radiation from the decay of ^{40}K and several radionuclides of the thorium and uranium series within the bulk sediment matrix plus cosmogenics[6], corrected for attenuation by the combined effects of water[7] and organic material[8]. Dose rate conversion factors[9], 0.033 ± 0.007 alpha efficiency[10,11], and grain-size dependent dose attenuation corrections[12] were applied.

Samples were collected both above and below the present groundwater table. We observed a strong trend in water content with grain size for both populations[13] and particularly for the saturated samples (Fig. S4); finer-grain samples showed higher water contents than coarser-grain samples. To obtain a robust relationship between median grain size (D50) and water content, we included in the analysis 34 samples of similar ages and burial depths from a related project, on which we will report elsewhere.

However, in situ water content is not always representative of the saturation conditions experienced over the full burial history of a sample. Groundwater is known to vary seasonally in Bangladesh due to the monsoon[14], and we sampled in March near the end of the dry period before monsoon onset. It was therefore reasonable to assume samples we identified as saturated at their time of collection were under saturation for the entirety of their burial history, and for these samples we used the measured in-situ water content. For the unsaturated samples, we estimated their saturated value using the linear relationship we identified between water content

and grain size (Fig. S4). We then calculated the likely time-averaged water content of unsaturated samples as the average of the in situ (representing the ~6 months of the dry season conditions) and calculated saturated (representing ~6 months of monsoon conditions in which the landscape is inundated) water contents. Five percent absolute uncertainty was added to water content estimate and 10% relative uncertainty was applied to the organic content estimate. Dose rate data are reported in Supplementary Data 3.

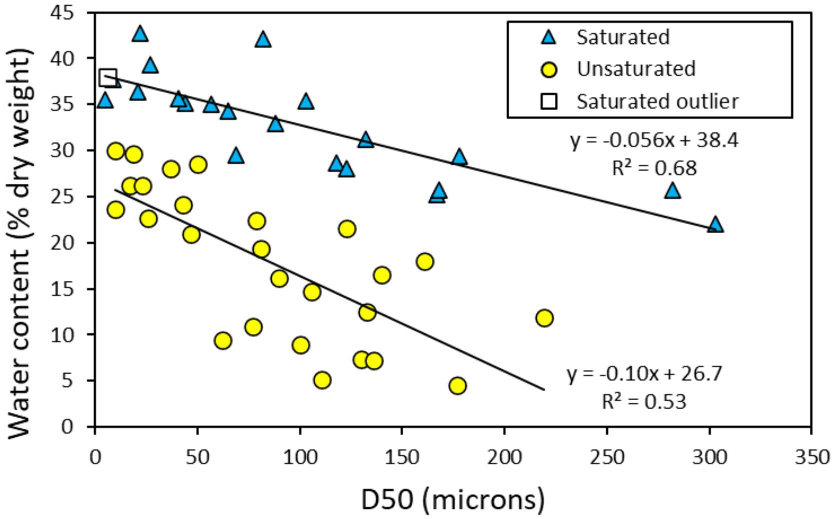

Fig. S4. Relationship between water content and median grain size (D50) for saturated and unsaturated samples (n=48) of this and another project. The saturated outlier, belonging to the other project, gave an unrealistically high water content and was not used to determine the linear fit of saturated samples; its estimated water content is shown here.

S4.3 Luminescence measurements, equivalent dose, paleodose, and age determinations. Luminescence measurements were made on one of four Risø TL/OSL DA-15 and DA-20 readers at NCL, providing dose rates of 0.145, 0.122, 0.110, or 0.104 ± 0.002 Gy/s to the silt and slightly higher (by ~8%) dose rates to the sand. Dosing times of regenerative points were corrected by machine so that each machine provided consistent doses to the samples across each protocol (Table S1). Aliquots were stimulated with blue LEDs (~470 nm) for quartz and infrared (~875 nm) LEDs for feldspar. All signals were detected through a 7.5 mm U340 filter. Luminescence age details are reported in Supplementary Data 3.

S4.3.1 Polymineral silt multiple-signal measurements. We began by measuring polymineral silt using a MS-SAR protocol[2,3], as this quickly gives insight into the burial dose range, degree of bleaching, and mineral sensitivities of each sample[3]. Our MS-SAR protocol (Table S1) measured four infrared stimulated (IR, then pIRIR) luminescence signals with increasing measurement temperatures (25, 90, 155, and 225 °C), a post-IR blue stimulated luminescence (referred to herein as pIR-BSL) signal at 125 °C, and a thermoluminescence signal. The IR signals, arising primarily from feldspar and measured for 100 s, were integrated over the first 2 s of the shine down curve and the backgrounds were integrated over the last 20 s. The pIR-BSL signal, arising primarily from quartz and measured for 20 s, was integrated over the first 0.5 s and an early background integrated over 0.5-1.8 s was subtracted to maximize the contribution of the readily

bleached fast component[15]. The thermoluminescence signal was integrated from 250 - 300 °C, with background was subtracted from 0-50 °C[16]. A mean ± standard error was used to determine the average equivalent dose (D_e) of each signal for each sample (e.g., $D_{e,IR25}$, $D_{e,pIRIR90}$, $D_{e,pIR\text{-}BSL}$, $D_{e,TL}$). From this, we determined that quartz silt was indeed the most viable material for dating our collection of samples, and we obtained a rough estimate of the D_e of each sample.

Table S1. MS-SAR protocol.

Treatment	Conditions
Dose	Natural, 10 Gy, 25 Gy, 0 Gy, 10 Gy
Preheat	250 °C, 30 s
IR 25	25 °C, 100 s
pIRIR 90	90 °C, 100 s
pIRIR 155	155 °C, 100 s
pIRIR 225	225 °C, 100 s
pIR-BSL 125	125 °C, 20 s
Test dose	10 Gy
Preheat	250 °C, 30 s
IR 25	25 °C, 100 s
pIRIR 90	90 °C, 100 s
pIRIR 155	155 °C, 100 s
pIRIR 225	225 °C, 100 s
pIR-BSL 125	125 °C, 20 s
TL	0-300 °C, 2 °C/s
OSL hot bleach	280 °C, 100 s

S4.3.2 Purified quartz silt measurements. A thermal transfer[17] test was performed to determine the optimal preheat temperature. Resulting values showed a rising trend with temperature and were less than 5 mGy for preheat temperatures up to 280ºC (Fig S5). The D_e values of purified quartz silt were obtained with one of two single-aliquot regenerative-dose (SAR) protocols[5,18] which were validated through dose recovery tests which returned values within 5% from unity (Fig S6). Both thermal transfer and dose recovery tests were conducted on three aliquots per sample for which there was sufficient material, and the data reported here include results from 34 samples from a related project which possessed similar attributes (see S4.2 – water content correction).

The SAR sequences used for equivalent dose estimation were tailored based on the anticipated paleodose of the samples, as judged by the D_e of the pIR-BSL signal obtained from the MS-SAR measurements signal ($D_{e,pIR\text{-}BSL}$), as well as the thermal transfer results. A SAR protocol employing three regenerative points (2.5, 5 and 10 Gy) and a 200 °C preheat was applied to all samples with $D_{e,pIR\text{-}BSL} < 5$ Gy (Table S2). The shape of the dose response curve for these low-dose samples could be accurately captured with three regenerative points constraining the range

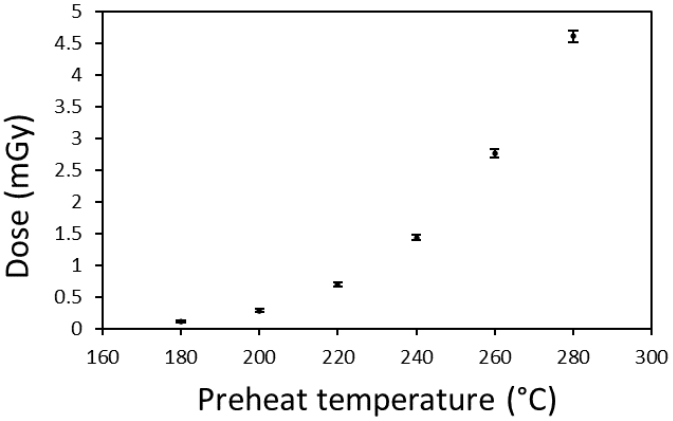

Fig. S5. Thermal transfer test results, with cumulative transferred dose shown in milligray. The data are an averaged from 87 aliquots.

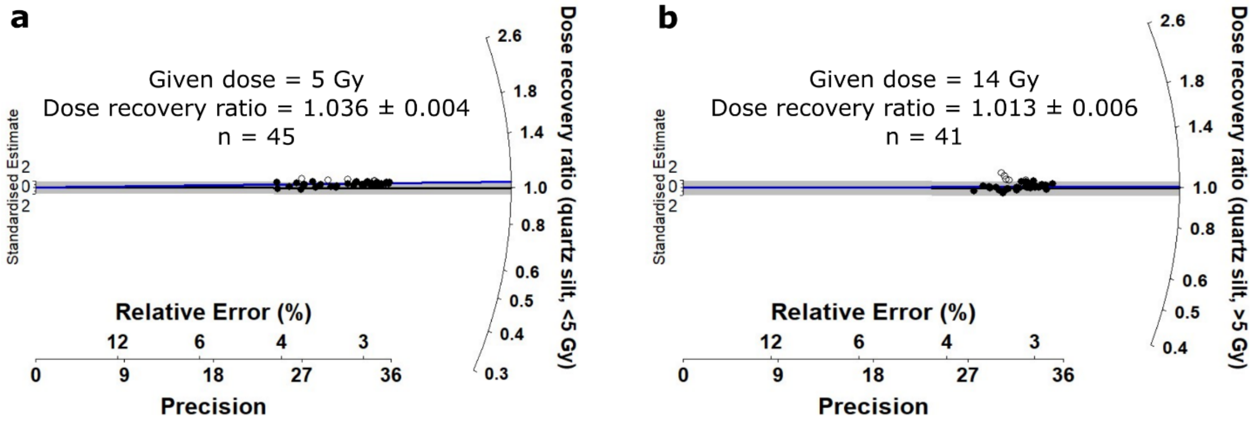

Fig. S6. Dose recovery results for (a) samples with $D_{e,pIR\text{-}BSL}$ <5 Gy and (b) $D_{e,pIR\text{-}BSL}$ >5 Gy. The black line indicates unity and the blue line indicates our results.

of interest. Furthermore, caution should be taken with the degree of preheating young samples, as small amounts of thermal transfer are relatively more significant than for older (higher burial dose) samples. A SAR protocol employing four regenerative points (5, 10, 20, 40 Gy) and a 240 °C preheat was applied to all samples with $D_{e,pIR\text{-}BSL}$ >5 Gy (Table S2), as greater regenerative points were needed to capture the relevant shape of the dose-response curve and thermal transfer associated with the higher preheat was deemed insignificant for these high-dose samples.

Aliquot acceptance criteria for all measurements (MS-SAR and standard SAR) applied a recycling ratio of 0.9-1.1, recuperation (relative to the largest regenerative dose) of less than 5%, an IR depletion ratio of less than 10% (Duller, 2003), and test dose error less than 20%, including uncertainties. Equivalent dose distributions of all samples are shown in Fig S7. As with the MS-SAR data, we calculated the paleodose of purified quartz silt using a mean ± standard error of all accepted aliquots. A mean is preferred to a central age model[19] because the central age model may preferentially weight higher dose aliquots due to lower relative uncertainty, and this can pose problems for dating very young Holocene deposits. OSL ages were determined as the quartz silt paleodose divided by the dose rate.

Table S2. SAR protocol to measure De of purified quartz silt for age estimation.

Treatment Group	Conditions $D_{e,pIR\text{-}BSL}$ < 5 Gy	Conditions $D_{e,pIR\text{-}BSL}$ > 5 Gy
Dosing	Natural, 2.5 Gy, 5 Gy, 10 Gy, 0 Gy, 5 Gy, 5 Gy (IR)	Natural, 5 Gy, 10 Gy, 20 Gy, 40 Gy, 0 Gy, 5 Gy, 5 Gy (IR)
Preheat	200 °C, 10 s	240 °C, 10 s
OSL	125 °C, 20 s	125 °C, 20 s
Test dose	5 Gy	5 Gy
Cutheat	180 °C, 10 s	220 °C, 10 s
OSL	125 °C, 20 s	125 °C, 20 s
Hot bleach	210 °C, 40 s	210 °C, 40 s

S4.4 Validity assessment. The OSL ages show excellent agreement in the undisturbed units including the paleochannel sand and mud at Magura, paleochannel sand at Ramdia, and capping mud of the seismite site. All support fluvial activity circa 2.6-2.5 ka. These ages are judged to be valid considering their internal agreement and suitable luminescence properties. Two samples (MA I-4 and RD III-2) were taken from soils at shallow depths (0.40-0.45 and 0.63-0.68 m respectively). The ages of these soil samples are interpreted as underestimates due to bioturbation within the soils which was evident in the near surface. While the bioturbated ages provide some minimum constraints, they are not helpful for pinpointing the time of river activity and so they are excluded from our assessment of avulsion time.

The sand dike (MA III-1) yields an age of 4.58 ± 0.31 ka, roughly two thousand years older than the mud it intrudes. Superposition therefore indicates that the sand dike age is an overestimate. The sands underlying the pond floor at the seismite site (MA VI-2, MA I-1, and MA VI-1) returned ages of 9.85 ± 2.08, 8.59 ± 0.51, and 8.00 ± 0.48 ka. While these ages agree within one sigma uncertainty, they most likely overestimate the age of sand deposition. Considering regional subsidence rates around 2 mm/yr[20] as well as the ^{14}C age of other shallow deposits within the upper ~5 m of the Ganges floodplain[21], an early Holocene depositional age for these sediments is highly unlikely. We suspect significant upwelling and mixing of older material from depth which likely introduced old grains to the samples. The samples therefore most likely give an average age of different, and deeper, materials rather than their own depositional age. This interpretation is supported by the mixed-source strontium signals of these sand samples as well as the sand dike result. Furthermore, substantial soil formation would be expected during a ~5000 year hiatus of sedimentation, and no soil formation was identified at the sand-mud contact (roughly 4 m below the land surface, Fig. 3a) of the seismite site.

Dose distributions are of little use for identifying disturbances (e.g., upwelling, bioturbation) or heterogeneous bleaching of silt aliquots due to the high degree of signal averaging among the many silt grains per disk. This underscores the importance for silt dating of using large datasets to check for internal consistency, vetting against external age constraints[2], and thoroughly considering and documenting the depositional context including through proxies such as degree of soil formation and geochemistry-informed provenance.

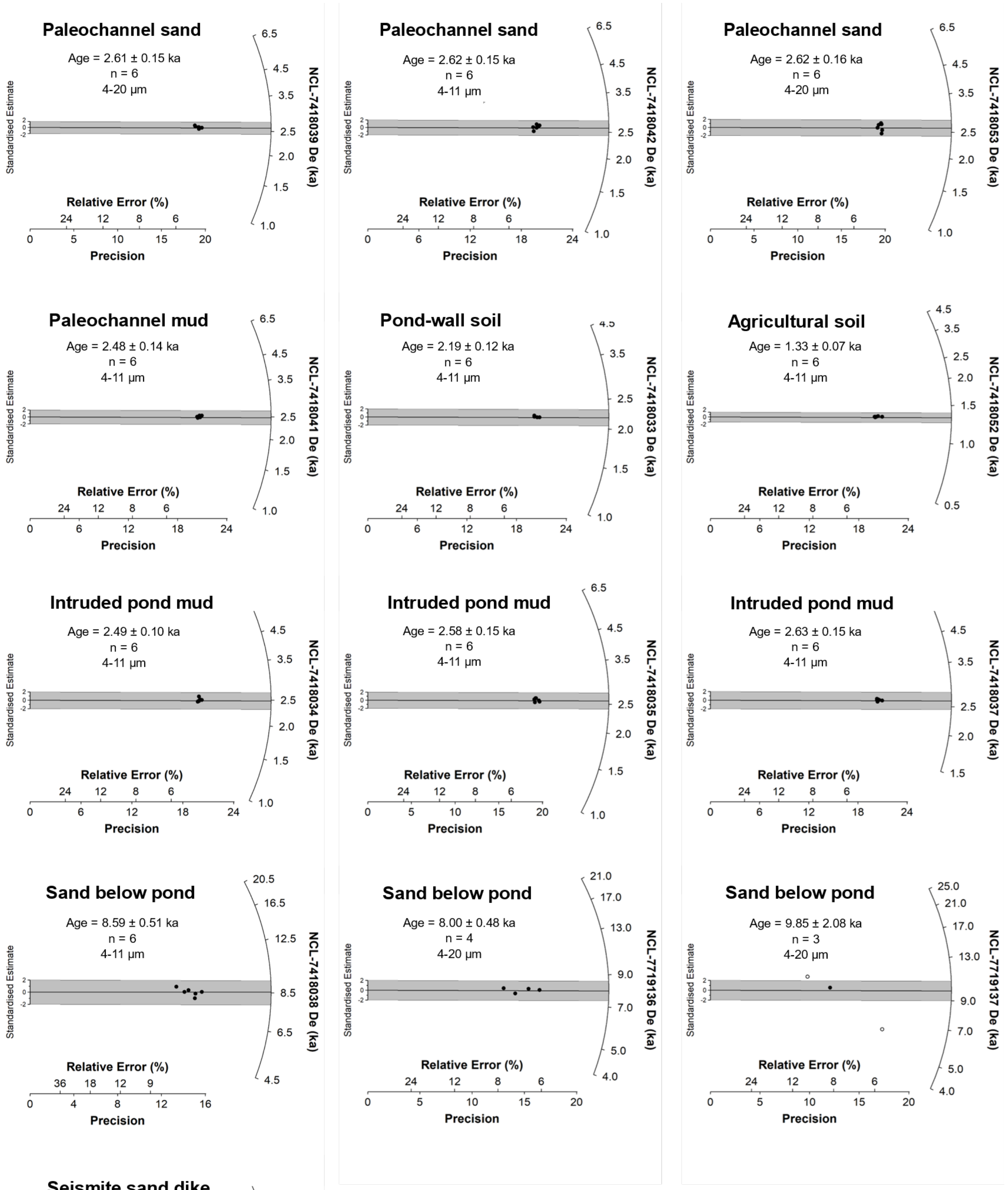

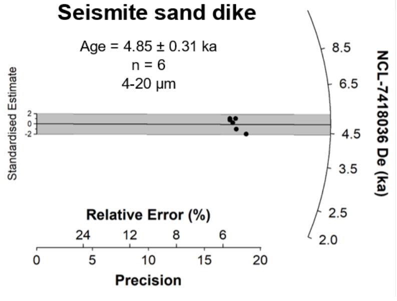

Fig. S7. Equivalent dose distributions (expressed as single-aliquot ages) of all samples dated for this project. Dose rate uncertainty is not included in this figure.

5. Tectonic setting and earthquake reconstruction

The orientation of the sand dikes is expected to be perpendicular to the maximum compressive stress. In the subducting Indian Plate, this is predominantly north-south, due to the influence of the Himalayan collision[22]. Focal mechanisms from the lower, subducting Indian Plate below the sediments of the delta, commonly show strike-slip faulting with downdip (east-west) extension and north-south compressional axes[23-25]. In the Indian plate both under the GBD and beneath the Indo-Burma Ranges the cold and rigid slab acts as a stress guide. Here, the extension direction and the subducting plate are sub-horizontal. Father east, the extension direction follows the plate as the plate bends and subducts below Burma.

Stress directions can vary with depth within the crust, particularly at subduction zones. In the upper plate east of the Indo-Burman deformation front, which is defined by fold structures observed in seismic and well data[26], the present-day stress field is oriented east-west (Fig. S8)[22]. This is expected, as orientation is perpendicular to the north-south folds of the Indo-Burman ranges.

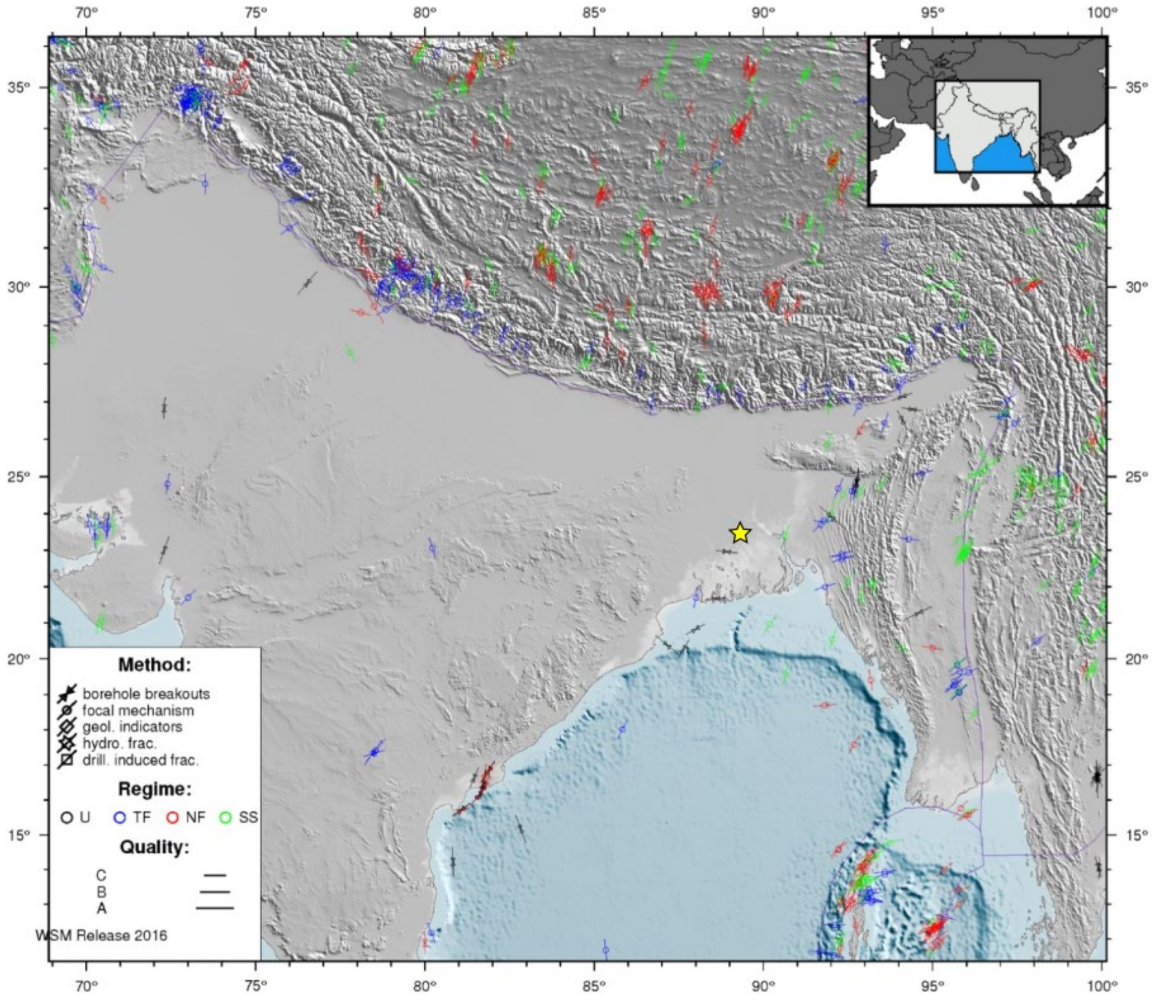

Fig. S8. Stress map of the Bengal basin and its bounding features, generated using the World Stress Map database[22]. Our study location is indicated by the yellow star.

Our field site is west of the deformation front and therefore north-south compression is expected in the Indian plate below the megathrust depth (~5.5 km). However, the shallower sediments may feel the east-west compression from the foldbelt. The sandy and relatively rigid upper layers in the delta may act as a guide for the stress responsible for the folding of the Indo-Burman ranges, and this stress could propagate west ahead of the deformation front. The sandy upper layers of fluvial and shallow marine sediments in the delta are stiffer than the more distal and finer sediment at depth in the delta[27]. The contact between these lithologies is thought to correlate with the megathrust detachment[28] and to extend westward across the delta beyond the deformation front, where it forms the top of overpressure[28]. Thus, the upper few kilometers at the seismite site may be subject to east-west compression. The shallow sediments may also experience increased east-west compression during earthquakes in the Indo-Burma ranges, but a sizable earthquake may be needed for east-west compression significant enough to cause fracturing at the seismite site.

In the Indo-Burman subduction zone, the detachment folds found near the deformation front transition to fault-propagation folds farther east beyond the thrust front. At least beyond the thrust front, the folds are underlain by the subduction megathrust capable of *M* 8+ earthquakes[29,30], and splay faults over the megathrust underlying the anticlines could be a source of *M* 7 earthquakes. It is unknown if the detachment folds between the deformation front and thrust front are sesimogenic. If the megathrust were to rupture, the most likely rupture zone source would be the downdip end of the locked portion of the fault[29,31]. These potential seismogenic parts of the Indo-Burman fold belt lie ~180-280 km from the sand-dike location. The orientations of the sand dikes and tectonic features are depicted in Fig. S9.

The locked portion of the Indo-Burman subduction margin is not known to have produced major fault ruptures, but the adjacent portion along the southeast Bangladesh and Arakan coast did rupture in 1762 to produce a major *M* 8.5+ earthquake[32,33]. This event is associated with the eruption of mud volcanoes in the Sitakund anticline in Bangladesh[34]. A recent study has linked the event to rapid subsidence and in-situ burial of salt kilns at the coast[35], presumably as a consequence of liquefaction and venting of underlying beds. The salt kilns are located along the

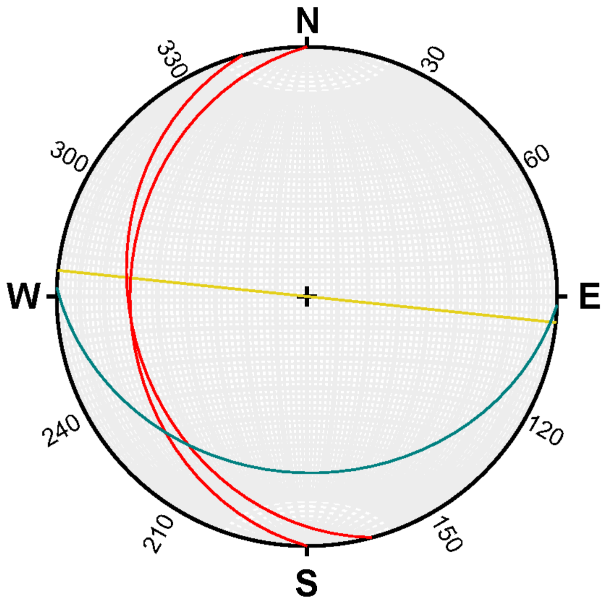

Fig. S9. Stereonet showing the directions of the seismite sand dikes (yellow), orientation of the Shillong Plateau basal thrust (blue), and orientation of the Indo-Burman ranges (red). Fault dips are 30°.

coast south of the sand-dike site, making them roughly equidistant from the Indo-Burman seismic zone with potential for liquefaction during a similar paleo-earthquake located farther north.

The sand-dikes lie 50-100 km east-southeast of the Indian plate Hinge zone (i.e., the craton-thinned crust transition), where there is a sharp transition in sediment thickness from 3-6 km to >12 km[36]. This sediment-to-bedrock basement interface could focus seismic energy to induce resonance and local intensification of ground motion in the central delta. The observed impacts could therefore have resulted from a smaller event that was amplified by basin resonance sequences[37-39]. Regardless, the surface damage was significant at a distance far from the nearest potential source. For example, the January 2017 *Mw* 5.7 Ambasa earthquake[40] sourced to Tripura, India, induced small sand-filled fractures (~1-cm thick) in a syncline of the Indo-Burman fold belt ~40-55 km from the origin[40]. Seismograph-recorded ground motion was 8-25 times stronger on the alluvial sediment compared to a nearby anticline (both sites 53-55 km from the epicentre)[41]. Similarly, the 1897 *M* 8+ earthquake originating from the north side of the Shillong Massif[42,43] caused heavy damage to buildings throughout the northern and central Bengal basin, with widespread liquefaction features noted within 20 km of the sand-dike location we study[44-46]. Many authors have linked a 1787 earthquake to initiating a 18th-19th century avulsion of the Brahmaputra River[34] although a more recent review of cartographic data only convincingly demonstrates that the avulsion initiated after the detailed 1764–73 Rennell maps and also shows that flow gradually diverted over at least ~ half a century[47].

The age of the sand dikes, at a few thousand years (i.e., ~2.5 ka), is also similar to the expected repeat times of large ruptures of the Burma megathrust (1-4 ky)[30]. This suggests that such a single rupture may be responsible both for the shaking that caused liquefaction and for the stress controlling the east-west orientation of the dikes. Generally, the static stress change from a rupture can affect seismicity in the upper crust up to 1.5 -2.0 rupture-dimensions away from that rupture. Alternatively, dike orientation could be conceivably controlled by dynamic stress during the passage of large surface waves. A way to test this hypothesis in the future may be to look for dikes generated on the delta by the 1897 earthquake, which is thought to have originated from a thrust fault at the Shillong anticlinorium absorbing north-south contraction[43]. If one large earthquake is sufficient to control clastic-dike directions, then those formed in the delta south of the 1897 Shillong event should be oriented north-south.

6. Development of a liquefaction-prone landscape

Satellite images reveal a growing number and cumulative area of dredge-fill construction sites near Khulna and Dhaka cities (n=49), Bangladesh (Fig. S10). The sites were mapped at 5-year intervals using Google Earth Engine[48] for the period 2003-2023. Such sites now cover almost 40 km^2 of reclaimed wetlands and floodplain, often supporting major private or public industrial infrastructure, including housing, transportation, and power facilities (Fig. S11).

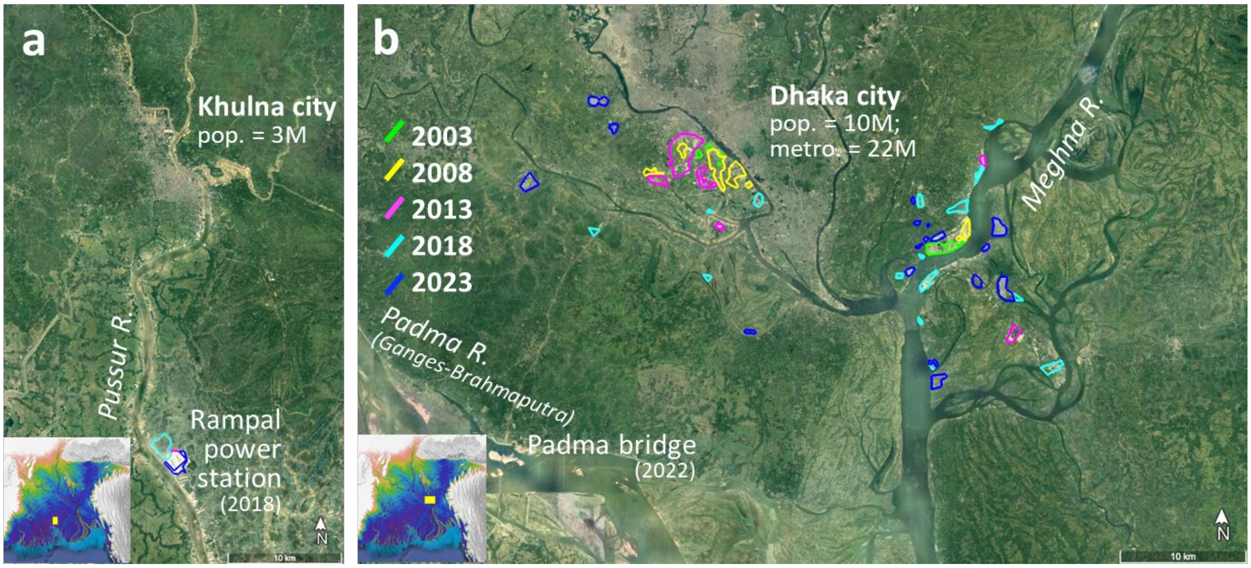

Fig. S10. Map of dredge-fill construction sites developed in areas of low-lying Holocene deltaplain (n=49) over the last 20 years near **a.** Khulna and **b.** Dhaka cities, Bangladesh. Images and data in panels a and b are from Google Earth Engine[48].

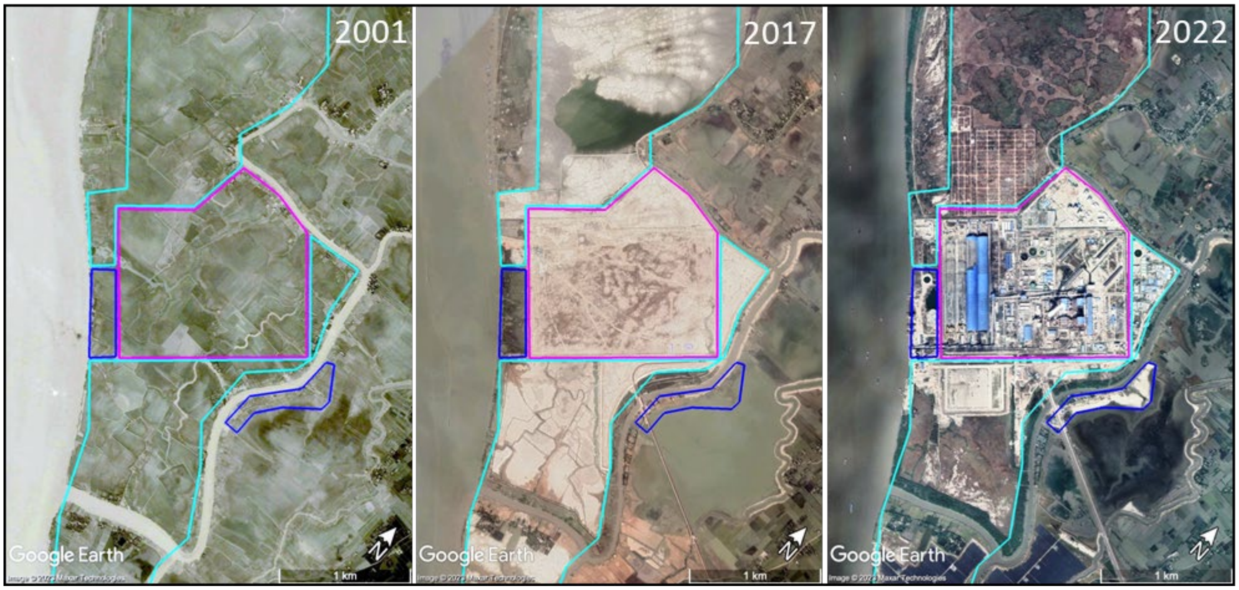

Fig. S11. Example dredge-fill construction site. This 742-hectare site is located 20 km south of Khulna and was developed for the 1320-megawatt, coal-fired Rampal power station. Location shown in panel 'a' of Fig. S10. Images and data are from Google Earth Engine[48].

Supplementary References

1 Goodbred, S. L. *et al.* Piecing together the Ganges-Brahmaputra-Meghna River delta: Use of sediment provenance to reconstruct the history and interaction of multiple fluvial systems during Holocene delta evolution. *Geol Soc Am Bull* **126**, 1495-1510, doi:10.1130/B30965.1 (2014).

2 Chamberlain, E. L. *et al.* Luminescence dating of delta sediments: novel approaches explored for the Ganges-Brahmaputra-Meghna Delta. *Quat Geochronol* **41**, 97-111, doi:10.1016/j.quageo.2017.06.006 (2017).

3 Reimann, T., Notenboom, P. D., De Schipper, M. A. & Wallinga, J. Testing for sufficient signal resetting during sediment transport using a polymineral multiple-signal luminescence approach. *Quat Geochronol* **25**, 26-36, doi:10.1016/j.quageo2014.09.002 (2015).

4 Duller, G. A. T. Distinguishing quartz and feldspar in single grain luminescence measurements. *Radiat Meas* **37**, 161-165, doi:10.1016/S1350-4487(02)00170-1 (2003).

5 Murray, A. S. & Wintle, A. G. The single aliquot regenerative dose protocol: potential for improvements in reliability. *Radiat Meas* **37**, 377-381, doi:10.1016/S1350-4487(03)00053-2 (2003).

6 Prescott, J. R. & Hutton, J. T. Cosmic-Ray Contributions to Dose-Rates for Luminescence and Esr Dating - Large Depths and Long-Term Time Variations. *Radiat Meas* **23**, 497-500, doi:Doi 10.1016/1350-4487(94)90086-8 (1994).

7 Aitken, M. J. *Thermoluminescence dating*. (Academic press, 1985).

8 Madsen, A. T., Murray, A. S., Andersen, T. J., Pejrup, M. & Breuning-Madsen, H. Optically stimulated luminescence dating of young estuarine sediments: a comparison with 210Pb and 137Cs dating. *Mar Geol* **214**, 251-268 (2005).

9 Guérin, G., Mercier, N. & Adamiec, G. Dose-rate conversion factors: update. *Ancient TL* **29**, 5-8 (2011).

10 Mauz, B., Packman, S. & Lang, A. The alpha effectiveness in silt-sized quartz: new data obtained by single and multiple aliquot protocols. *Ancient TL* **24**, 47-52 (2006).

11 Lai, Z. P., Zöller, L., Fuchs, M. & Brückner, H. Alpha efficiency determination for OSL of quartz extracted from Chinese loess. *Radiat Meas* **43**, 767-770 (2008).

12 Mejdahl, V. Thermoluminescence Dating - Beta-Dose Attenuation in Quartz Grains. *Archaeometry* **21**, 61-72, doi:DOI 10.1111/j.1475-4754.1979.tb00241.x (1979).

13 Nelson, M. S. & Rittenour, T. M. Using grain-size characteristics to model soil water content: application to dose-rate calculation for luminescence dating. *Radiat Meas* **81**, 142-149 (2015).

14 Steckler, M. S. *et al.* Modeling Earth deformation from monsoonal flooding in Bangladesh using hydrographic, GPS, and Gravity Recovery and Climate Experiment (GRACE) data. *Journal of Geophysical Research: Solid Earth* **115** (2010).

15 Cunningham, A. C. & Wallinga, J. Selection of integration time intervals for quartz OSL decay curves. *Quat Geochronol* **5**, 657-666, doi:10.1016/j.quageo.2010.08.004 (2010).

16 Rink, W. J. Thermoluminescence of quartz and feldspar sand grains as a tracer of nearshore environmental processes in the southeastern Mediterranean Sea. *J Coastal Res* **19**, 723-730 (2003).

17 Truelsen, J. L. & Wallinga, J. Zeroing of the OSL signal as a function of grain size: investigating bleaching and thermal transfer for a young fluvial sample. *Geochronometria* **22**, 1-8 (2003).

18 Murray, A. S. & Wintle, A. G. Luminescence dating of quartz using an improved single-aliquot regenerative-dose protocol. *Radiat Meas* **32**, 57-73, doi:Doi 10.1016/S1350-4487(99)00253-X (2000).

19 Galbraith, R. F., Roberts, R. G., Laslett, G. M., Yoshida, H. & Olley, J. M. Optical dating of single and multiple grains of quartz from Jinmium rock shelter, northern Australia: Part I, experimental design and statistical models. *Archaeometry* **41**, 339-364 (1999).

20 Steckler, M. S. *et al.* Synthesis of the distribution of subsidence of the lower Ganges-Brahmaputra Delta, Bangladesh. *Earth-Science Reviews* **224**, 103887 (2022).

21 Raff, J. L. *et al.* Sediment delivery to sustain the Ganges-Brahmaputra delta under climate change and anthropogenic impacts. *Nature communications* **14**, 2429 (2023).

22 Heidbach, O. *et al.* The World Stress Map database release 2016: Crustal stress pattern across scales. *Tectonophysics* **744**, 484-498 (2018).

23 Fadil, W. *et al.* Active Faults Revealed and New Constraints on Their Seismogenic Depth from a High-Resolution Regional Focal Mechanism Catalog in Myanmar (2016–2021). *Bulletin of the Seismological Society of America* **113**, 613-635 (2023).

24 Mon, C. T. *et al.* Insight into major active faults in central Myanmar and the related geodynamic sources. *Geophysical Research Letters* **47**, e2019GL086236 (2020).

25 Ni, J. F. *et al.* Accretionary tectonics of Burma and the three-dimensional geometry of the Burma subduction zone. *Geology* **17**, 68-71 (1989).

26 Betka, P. M. *et al.* Slip-partitioning above a shallow, weak décollement beneath the Indo-Burman accretionary prism. *Earth Planet Sc Lett* **503**, 17-28 (2018).

27 Steckler, M. S., Akhter, S. H. & Seeber, L. Collision of the Ganges-Brahmaputra Delta with the Burma Arc: Implications for earthquake hazard. *Earth Planet Sc Lett* **273**, 367-378, doi:10.1016/j.epsl.2008.07.009 (2008).

28 Zahid, K. M. & Uddin, A. Influence of overpressure on formation velocity evaluation of Neogene strata from the eastern Bengal Basin, Bangladesh. *J Asian Earth Sci* **25**, 419-429 (2005).

29 Oryan, B. *et al.* New GNSS and geological data from the Indo-Burman subduction zone indicate active convergence on both a locked megathrust and the Kabaw Fault. *Journal of Geophysical Research: Solid Earth*, e2022JB025550 (2023).

30 Vorobieva, I., Gorshkov, A. & Mandal, P. Modelling the seismic potential of the Indo-Burman megathrust. *Sci Rep-Uk* **11**, 21200 (2021).

31 Steckler, M. S. *et al.* Locked and loading megathrust linked to active subduction beneath the Indo-Burman Ranges. *Nat Geosci* **9**, 615 (2016).

32 Wang, Y. *et al.* Permanent upper plate deformation in western Myanmar during the great 1762 earthquake: Implications for neotectonic behavior of the northern Sunda megathrust. *Journal of Geophysical Research: Solid Earth* **118**, 1277-1303 (2013).

33 Cummins, P. R. The potential for giant tsunamigenic earthquakes in the northern Bay of Bengal. *Nature* **449**, 75-78 (2007).

34 Akhter, S. H. Earthquakes of Dhaka. *Environment of Capital Dhaka—plants wildlife gardens parks air water and earthquake. Asiatic Society of Bangladesh, Dhaka*, 401-426 (2010).

35 Hanebuth, T. J. *et al.* Stepwise, earthquake-driven coastal subsidence in the Ganges–Brahmaputra Delta (Sundarbans) since the eighth century deduced from submerged in situ kiln and mangrove remnants. *Natural Hazards*, 1-28 (2022).

36 Singh, A. *et al.* Crustal structure and tectonics of Bangladesh: New constraints from inversion of receiver functions. *Tectonophysics* **680**, 99-112 (2016).
37 Rial, J. A., Saltzman, N. G. & Ling, H. Earthquake-induced resonance in sedimentary basins. *American Scientist* **80**, 566-578 (1992).
38 Wirth, E. A. *et al.* Source-dependent amplification of earthquake ground motions in deep sedimentary basins. *Geophysical Research Letters* **46**, 6443-6450 (2019).
39 Pratt, T. L. *et al.* Amplification of seismic waves by the Seattle basin, Washington State. *Bulletin of the Seismological Society of America* **93**, 533-545 (2003).
40 Debbarma, J., Martin, S. S., Suresh, G., Ahsan, A. & Gahalaut, V. K. Preliminary observations from the 3 January 2017, MW 5.6 Manu, Tripura (India) earthquake. *J Asian Earth Sci* **148**, 173-180 (2017).
41 McHugh, C. M. *et al.* in *AGU Fall Meeting Abstracts.* T23C-0381.
42 England, P. & Bilham, R. The Shillong Plateau and the great 1897 Assam earthquake. *Tectonics* **34**, 1792-1812 (2015).
43 Subedi, S. & Hetényi, G. Precise Locating of the Great 1897 Shillong Plateau Earthquake Using Teleseismic and Regional Seismic Phase Data. *The Seismic Record* **1**, 135-144 (2021).
44 Ambraseys, N. & Bilham, R. Reevaluated intensities for the great Assam earthquake of 12 June 1897, Shillong, India. *Bulletin of the Seismological Society of America* **93**, 655-673 (2003).
45 Oldham, R. D. Report on the great earthquake of 12th June 1897. 1-379 (Calcutta, 1899).
46 Hough, S. E., Bilham, R., Ambraseys, N. & Feldl, N. Revisiting the 1897 Shillong and 1905 Kangra earthquakes in northern India: Site response, Moho reflections and a triggered earthquake. *Curr Sci India*, 1632-1638 (2005).
47 Bandyopadhyay, S., Das, S. & Kar, N. S. Avulsion of the Brahmaputra in Bangladesh during the 18th–19th century: A review based on cartographic and literary evidence. *Geomorphology* **384**, 107696 (2021).
48 Gorelick, N. *et al.* Google Earth Engine: Planetary-scale geospatial analysis for everyone. *Remote sensing of Environment* **202**, 18-27 (2017).
