# Supplementary material for: Cascading hazards of a major Bengal basin earthquake and abrupt avulsion of the Ganges River
Source: Nat Commun. 2024 Jun 17;15:4975. doi: 10.1038/s41467-024-47786-4 (PMC11183210; doi:10.1038/s41467-024-47786-4)
Supplement: Supplementary file 3 — Description of Additional Supplementary Files [file 41467_2024_47786_MOESM3_ESM.pdf]

Description of Additional Supplementary Files

File Name: Supplementary Data 1 – 3

Supplementary Data 1
Description: Description of boreholes/exposures including locality, borehole/exposure number, date, coordinates, depth, surface elevation, OSL samples, and landscape.

Supplementary Data 2
Description: OSL sample information including sample name, Netherlands Centre for Luminescence dating (NCL) code, Vanderbilt University (VU) code, borehole/exposure.

Supplementary Data 3
Description: OSL age details including sample name, NCL code, VU code, analysed grain size fraction, number of accepted aliquots, paleodose, cosmogenic dose, uranium content, thorium content, potassium content, water and organic content as percent dry weight, dose rate, and age relative to 2020.
